# siRNA Design to Silence the 3′UTR Region of Zika Virus

**DOI:** 10.1155/2020/6759346

**Published:** 2020-08-02

**Authors:** María Perez-Mendez, Paola Zárate-Segura, Juan Salas-Benito, Fernando Bastida-González

**Affiliations:** ^1^Laboratorio de Medicina Traslacional, Escuela Superior de Medicina, Instituto Politécnico Nacional, St. Salvador Díaz Mirón Esquina Plan de San Luis, Santo Tomas, Miguel Hidalgo, CDMX 11340, Mexico; ^2^Laboratorio de Biomedicina Molecular 3, Escuela Nacional de Medicina y Homeopatía, Instituto Politécnico Nacional, Guillermo Massieu Helguera 239, La Escalera-Ticomán, Gustavo A. Madero, CDMX 07329, Mexico; ^3^Laboratorio de Biología Molecular, Laboratorio Estatal de Salud Pública del Estado de México, Paseo Tollocan s/n, La Moderna de la Cruz, EDOMEX, Toluca, 50180, Mexico

## Abstract

The disease caused by the Zika virus (ZIKV) has positioned itself as one of the main public health problems in Mexico. One of the main reasons is it causes microcephaly and other birth defects. The transmission of ZIKV is through *Aedes aegypti* and *Ae. albopictus* mosquitoes, which are found in a larger space of the national territory. In addition, it can also be transmitted via blood transfusion, sexual relations, and maternal-fetal route. So far, there are no vaccines or specific treatments to deal with this infection. Currently, some new therapeutics such as small interfering RNAs (siRNAs) are able to regulate or suppress transcription in viruses. Therefore, in this project, an *in silico* siRNA was designed for the 3′UTR region of ZIKV via bioinformatics tools. The designed siRNA was synthesized and transfected into the C6/36 cell line, previously infected with ZIKV in order to assess the ability of the siRNA to inhibit viral replication. The designed siRNA was able to inhibit significantly (*p* < 0.05) ZIKV replication; this siRNA could be considered a potential therapeutic towards the disease that causes ZIKV and the medical problems generated.

## 1. Introduction

In 1947 in the Zika Forest, Uganda, Zika virus was isolated for the first time. The spread of ZIKV was not evident until late 2015 and early 2015 when it arrived to the Americas, specifically to Brazil [1, 2]. Later, the virus spread throughout the continent, arriving to Mexico in 2015, so far there are 12,936 confirmed cases of ZIKV from 2015 to epidemiological week 5 of 2020, which 7,135 cases correspond to pregnant women, also 54 confirmed cases of congenital syndrome associated with ZIKV, 10 deaths; and 34 confirmed cases of Guillain-Barre syndrome associated with ZIKV [3].

The main mechanism of transmission of ZIKV is through the bite of mosquitoes of the *Aedes* species, specifically *Ae. aegypti* and *Ae. albopictus*, as well as blood transfusion, maternal-fetal transmission, and sexual intercourse, causing ZIKV disease, which is asymptomatic in 80% of cases, but is associated to serious complications such as Guillain-Barre syndrome, microcephaly, and other birth defects [2].

ZIKV is structured by a polyprotein that encodes 3 structural proteins: capsid (C), membrane precursor (prM), and envelope (E), in addition to 7 nonstructural proteins (NS1, NS2A, NS2B, NS3, NS4A, NS4B, and NS5) flanked by two untranslated regions (UTR): 5′UTR and 3′UTR, emphasizing the 3′UTR region plays an important role in the viral genome replication and pathogenic processes [4].

Until now, there are no vaccines or antiviral agents against ZIKV disease [5]. RNA interference (RNAi) has been positioned as therapeutic tool due to the fact that it has a mechanism to regulate or suppress transcription, within the interfering RNAis there are the small interfering RNAs (siRNAs) of a size of ~21 nt capable of performing gene silencing [6]. So far, progress has been made in the deliberation of siRNA as therapeutic agents to combat different diseases as well as viruses [7].

In the present work, a siRNA targeting the 3′UTR region of ZIKV was designed to reduce or inhibit viral replication and cytopathic effect of the virus. It has been shown that siRNAs can perform specific gene silencing of functional sequences.

## 2. Materials and Methods

### 2.1. siRNA Design and Validation

Over hundred and twenty-eight sequences from the complete viral genome or the 3′UTR region were obtained from the NCBI database. They belong to African, American, and Asian linages of ZIKV. Sequences were aligned trough MEGA 7 and Jalview (version 2.9.0b2) program in order to obtain the 3′UTR region and generate groups; then a consensus sequence of each group was determined. Consensus sequences were submitted to 8 different bioinformatics servers (Sidirect 2.0 [8], InvivoGen [9–11], AsiDesigner [12], DSIR [13], OligoWalk [14], Sfold [15], VIRsiRNA [16] and i-Score Designer [17]), which have specific rules and/or criteria for the efficient design of siRNAs *in silico*. Selected siRNA was the one which was homolog to the consensus sequences and selected by the eight servers.

Afterwards, validation was made, consisting in calculating the folding energy (*Δ*GF) of the siRNA by its secondary structure using the Mfold web [18] and RNAstructure servers [19]. To calculate the binding energy (*Δ*GB) between the siRNA and its target sequence, the server RNAcofold program [20] was used; also, the breaking energy (*Δ*GR) of the 3′UTR region where the target sequence is found, was calculated with the Sfold program [15]. Finally, the siRNA was aligned with the human genome through blast tool (NCBI) to avoid off-targets (87 siRNA 3D model design and validation).

Obtained siRNA was modelled by its secondary structure via Vienna format; subsequently, it was submitted to the RNAComposer server [21, 22]. The model obtained was validated with the MOLProbity server [23], analyzing all-atom contacts and geometry, RNA sugar puckers, RNA backbone conformations, hydrogen bonds, and Van der Waals force. Wrong RNA sugar puckers and RNA backbone conformations were fixed manually with RNA rotator Tool with KiNG 2.22 [24] and reevaluated with MOLProbity to find the most accurate 3D model.

### 2.2. Cell Culture

C6/36-HT cell line was used. Cells were maintained in minimum essential medium (MEM) supplemented with vitamins, 10% fetal bovine serum (FBS), 0.034% sodium bicarbonate, streptomycin (100 *μ*g/mL), and penicillin (100 U/mL). They were incubated at 35°C without CO2.

### 2.3. Viral Replication

The viral strains of ZIKV, dengue virus 1 (DENV-1), and chikungunya virus (CHIKV) were kindly donated by the National School of Medicine and Homeopathy. A multiplicity of infection (MOI) of 0.001 was used. C6/36-HT cells were infected for 1 hour before siRNA transfection.

### 2.4. Experimental Groups

Five different groups were carried out for the study. The assay was performed in a 12-well plate, in triplicate for the 3 viruses (ZIKV, DENV-1, and CHIKV). The following are the five groups: group 1—C6/36 cells infected with either one of the virus as positive control; group 2—C6/36 cells infected with either one of the virus adding Lipofectamine® 2000 Reaction Reagent (Thermo Fisher Scientific, USA); group 3—C6/36 cells infected with either one of the virus and transfected with the siRNA at a concentration of 0.5 *μ*g; group 4—C6/36 cells infected with either one of the virus and transfected with the siRNA at a concentration of 1 *μ*g; and group 5—C6/36 cells infected with either one of the virus and transfected with the siRNA at a concentration of 2 *μ*g. The groups were compared to measure the efficiency of the siRNA to inhibit viral replication. Negative control with noninfected cells was also carried out in triplicate.

### 2.5. Viral Infection and Transfection

C6/36 cells were seeded in a 12-well plate (5 × 10^5^ cells/well) adding 1 mL of medium and incubated overnight in the conditions previously described. The next day, at a confluence, ~80% were infected with ZIKV, DENV, or CHIKV at a MOI of 0.001. The infection was carried out for 1 h with gentle shaking at 37°C. Then, the virus was removed, cells were washed with 1 mL of phosphate buffer saline (PBS) + 0.5%FBS, and 1 mL of medium was added to each well. Immediately, the transfection was performed with the 3 concentrations of siRNA (0.5 *μ*g, 1 *μ*g, and 2 *μ*g) via Lipofectamine® 2000 Reaction Reagent (Thermo Fisher SCIENTIFIC, USA) following the manufacturer's instructions. C6/36 cells were incubated for 3 days as indicated above.

### 2.6. Viral RNA Extraction

Viral RNA extraction was performed 3 days post infection (dpi), using QIAmp® Viral RNA Mini Kit (QIAGEN, Germany) following the manufacturer's protocol.

### 2.7. RT-qPCR Viral Detection

Viral detection was performed with a Trioplex [25], for the detection of the 3 viruses.

The relative viral quantity of each group was represented with the CT values; the change between each group compared to the control was calculated using *Δ*CT (CTgroup − CTcontrol).

### 2.8. Statistical Analysis

Student's *t*-test was used to compare the control group with the 3 siRNA concentration.

A *p* ≤ 0.05 was considered significant. Statistical analysis was done with Sigma Plot (version 14.0) program.

## 3. Results and Discussion

**Figure 1:**
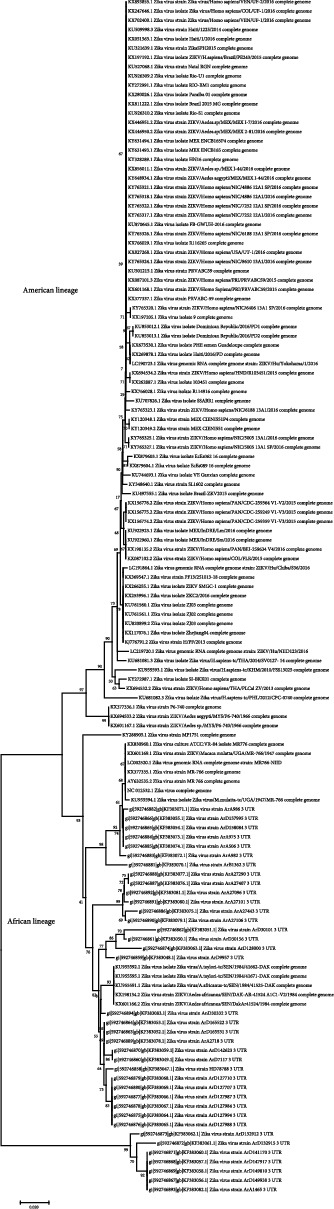
Phylogenetic tree of the 128 sequences of the 3′UTR region. Asian and African lineages are shown.

ZIKV, discovered in 1947, has two lineages: African and Asian [26]. A total of 128 sequences from the complete genome or from the 3′UTR region of both lineages of Zika virus were obtained from the NCBI database. Sequences were aligned, and the 3′UTR region was obtained via MEGA 7. A phylogenetic tree (Figure 1) was made through MEGA 7. The two lineages can be distinguished, with also a subgroup within the African strain [27]. In the first demarcation sequences, Asia and America strains can be distinguished; thus, circulating ZIKV in America is of Asian origin. Phylogenetic and molecular clock analyses were performed where it is shown that a single introduction of ZIKV to America was made, estimated in May 2013 and December 2013, which weakens the hypothesis that the 2014 Soccer World Cup (June 2 to July 13, 2014) and the Va'a canoe event (August 12-17, 2014) were the origin of the epidemic in America [28]. A total of 10 groups were made, and their consensus sequences were obtained through MEGA 7. These consensus sequences were submitted to the 8 siRNA bioinformatics servers. A final siRNA with a length of 21 nt was generated, homologous in the 8 servers and in the 10 consensus sequences.

siRNA validation is important to ensure efficient silencing of the given area in of the viral genome. The Reynolds rules [29] are contemplated in 3 of the servers used as well as the other number of rules or parameters given in each server to guarantee a functional siRNA. The first validation was G/C content; designed siRNA had a G/C content of 42.1%; according to the specified rules, an optimal G/C content between 36% and 52% is adequate [29], given that if it has a higher G/C content, it will decrease the splitting of the dsRNA by the helicase associated with the RISC complex and could generate a secondary structure that prohibits the union of the recognition and hybridization between the target and the siRNA and the efficient binding of Ago2 [30]; siRNA also showed a low stability (uracil) in the 5′UTR of the antisense chain; it has been shown that a low internal stability of the dsRNA at the end of the 5′UTR side of the antisense chain is a prerequisite for effective silencing and efficient entry of the chain mentioned to the RISC [17, 30].

**Figure 2:**
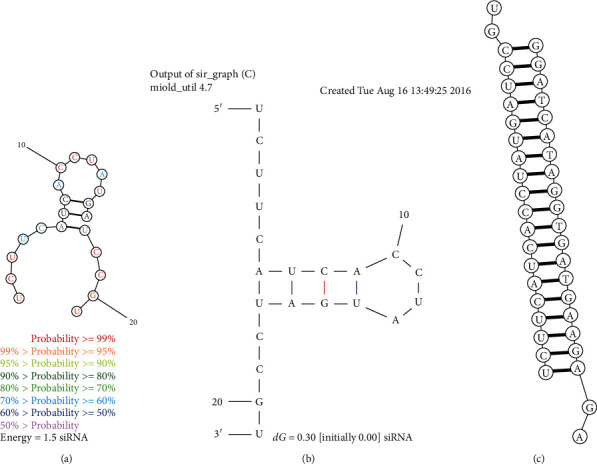
siRNA validation. (a) Secondary structure of the siRNA by Mfold web server; (b) secondary structure of the siRNA by RNAstructure web server; (c) siRNA target interaction by RNAcofold program.

The second validation was focused on the Minimum Free Energy (MFE) for folding (*Δ*GF); it is obtained by means of the secondary structure of the siRNA, which plays an important role in its efficiency. There is evidence suggesting that the secondary structure can cause an inhibition of RNAi and has influence into the efficiency of the siRNA-target interaction [31, 32]. Two secondary structures were given by the two servers resulting in two *Δ*GF:   1.5 kcal/mol (Figure 2(a)) and 0.3 kcal/mol (Figure 2(b)).

The RNA molecule also must have stability; assessments have been carried out demonstrating that molecules with positive or near zero energies can be more accessible to the target site and can have a greater power in the binding to carry out gene silencing [33]; both values represent positive energies. Posteriorly, energy between the target and the siRNA was calculated resulting in a *Δ*GB of 34.7 kcal/mol (Figure 2(c)); the interaction between siRNA and target generates binding energy, so a favorable thermodynamic organization of the duplex is a prerequisite for silencing [34]; it has been shown that high negative (*Δ*GB) values reflect a stable internal structure; the silencing efficiency correlates with the binding energies of the siRNA, and its target [33, 35] results indicate a high negative union.

The next steps consisted in the rupture energy (*Δ*GR); *Δ*GR obtained was between 6.8 and 9.65 kcal/mol, due to two ZIKV lineages and 10 SC obtained; the breaking energy *Δ*GD was calculated for each one. The rupture energy refers to the accessibility of the target. Although the designed siRNA has the necessary rules and characteristics, it will not necessarily be functional if it cannot be efficiently joined with a highly structured target; a ΔGr > −10 kcal/mol increases the efficiency of silencing [36]. Therefore, the different conformations of the CS of the 3′UTR can be efficiently accessible to the siRNA. Finally, the siRNA was BLAST with human genome throwing a similarity > 35%; the design of a siRNA of 21 nt and its alignment with the human genome is essential to prevent the activation of the immune response by dsRNA-dependent protein kinase (PKR), which was identified as a sensor for dsRNA > 33 pb; MDA5 also can detect dsRNA (~21 nt) when a sequence dependent of the human genome is identified inducing the activation of interleukin-6 (IL-6) and Tumor Necrosis Factor-*α* (TNF-*α*) [37].

**Figure 3:**
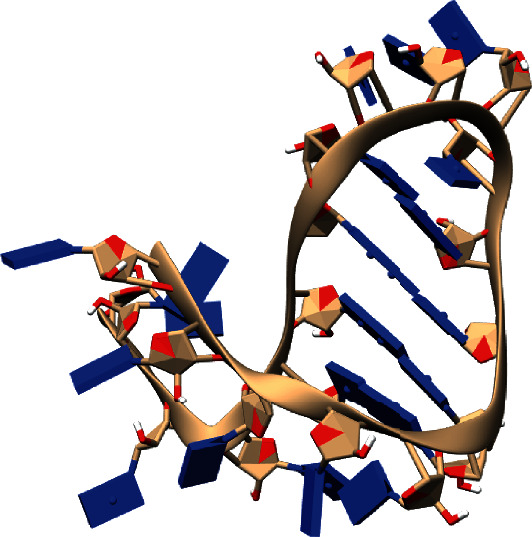
Tertiary structure of the siRNA designed by RNAComposer.

After siRNA validation, 3D structure of the siRNA was obtained (Figure 3) via the RNAComposer server and validated with MOLProbity. The validation results were as follows: nonclash score atoms were shown, 3 bad backbone conformations were identified after manual correction was done with an RNA rotator with KiNG 2.22 tool, goal is a percent ≤ 5%, also, nonbad bones or bad angles were identified, and nonbad sugar puckers were identified. In contrast with proteins, just a small amount of RNAs have been evaluated by X-ray crystallography, RMN spectroscopy, and cryoelectronic microscopy (cryo-EM) and added to base date; thus, a homology model cannot be employed with the siRNA designed, that is why prediction methods have been designed to provide secondary and tertiary structures of RNA [21]; the success of the algorithm prediction is based on experimental data, evolutive information, and the experts to selection and positioned tertiary noncanonical characteristics in the final models [38]; importance of predicting these tertiary structures fall in showing how the 3D structure of the RNAi is shown and how the structure is revealed when joining its target [39]; the structure designed was the most suitable to the reality although some penalties in the modelled designed are observed.

**Figure 4:**
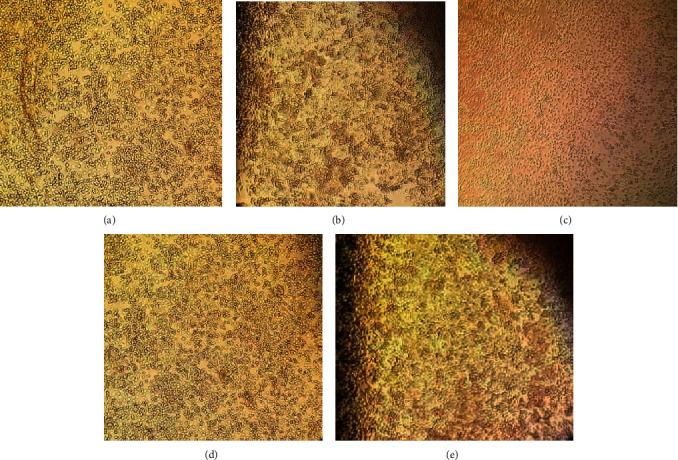
C6/36 cell line 3 dpi infected with ZIKV at MOI of 0.001. (a) C6/36-infected cells 3 dpi; (b) C6/36-infected cells 3 dpi transfected with Lipofectamine 2000; (c) C6/36-infected cells transfected with 0.5 *μ*g of siRNA; (d) C6/36-infected cells transfected with 1 *μ*g of siRNA; (e) C6/36-infected cells transfected with 2 *μ*g of siRNA.

After validation of the siRNA, it was synthesized and transfected into C6/36 cells infected with ZIKV, DENV, or CHIKV; ZIKV is deposited in the epidermis and dermis, through the bite of a mosquito. Due to the presence of fibroblasts, keratinocytes, and dendritic cells, the virus can have easy access to the host through cellular receptors, which causes the entry of viral RNA to the cytoplasm [40], being an accessible target for the siRNA since the machinery to perform the silencing—RISC complex—and generate the siRNA from a dsRNA is found in the cytoplasm [41]. Cell line C6/36 has a Dicer (Dcr2) deficient, although it can generate a functional RNA-induced silencing complex (RISC). The defective RNAi pathway of C6/36 cells produces the inability to the cell line to have an effective antiviral defense against virus causing a greater viral infection than other cell lines. In addition, it is essential that the precursor of the siRNA has 21 nt long due to the fact that C6/36 cells do not have the ability to generate long dsRNA siRNAs; however, there is a reduction in the expression of Dcr2 in the C6/36 cell line compared with its simile the Aag2 cell line from *Aedes aegypti*; the magnitude of reduction does not seem to be enough to consider a complete lack of Dicer activity. It has also been suggested that this cell line has a backup, using the piwi-RNA (piRNA) pathway as a compensation for the processing of exogenous double stranded RNA (dsRNA) [42], indicating that efficient gene silencing can be performed with this cell line 3 dpi and transfection a nonuniform and lower growth in cells infected and transfected with siRNA which was showed (Figure 4); it has been previously described that C6/36 cells do not show an important cytopathic effect when infected with Zika virus [43]. In the other hand, transfection was elaborated through cationic liposomes; these have emerged as a possible promising alternative of therapy instead of viral vectors thanks to their safety and versatility; in addition, they are effective in mediating the delivery of polynucleotides; numerous trials have been reported *in vivo* in the literature of these molecules focusing on pharmacokinetics and biodistribution, as well as their toxicity and immunogenicity, and in clinical trials, although the efficacy of this delivery compared to vectors is still far away [44].

RNA was extracted from the cells, and the presence of the viral genome was determined by RT-qPCR (Table 1); the first group and the second group obtained a CT value of 22.33 ± 0.43, the 3rd with 0.5 *μ*g of siRNA (CT: 24.56 ± 0.77), the 4th with 1 *μ*g of siRNA (CT: 28.24 ± 0.34), and the last group with 2 *μ*g of siRNA (CT: 33.73 ± 1.82) (Table 1). A statistical significance (*p* < 0.05) between the control group and the siRNA at 1 *μ*g and the siRNA at 2 *μ*g was obtained using Student's *t*-test.

At the present, there are not *in vitro* or *in vivo* assays of siRNAs raised against Zika virus; however, there are other studies carried out with other flaviviruses. A designed siRNA targeting the membrane glycoprotein (prM) precursor gene of DENV showed an increase in Ct, from 14.56 to 19.91, with a statistical significance (*p* < 0.05) and a transfection efficiency of 66% with the siRNA at 1 *μ*g using the HiPerFect Transfection reagent in C6/36 cells transfected before infection with DENV-1 at 100 TCID50 [45]. The design of different siRNAs against the genome of each of the Dengue virus serotypes (DENV1-4), at an amount of 1 to 10 *μ*M transfected with 1 *μ*L of RNAiMAX in Huh7 cells, which were subsequently infected with the virus at an MOI of 0.2 or 0.5 for 2 h, resulted in a 10- to 100-fold reduction in viral infection corresponding to a marked reduction in viral protein levels. In addition, an *in vivo* assay was performed in intravenously infected AG129 mice with 109 genomic equivalents (equivalent to 20,000 PFU, measured by plaque assay in BHK cells); a mixture of Silencer In Vivo Ready siRNA (10 mg/kg) and Invivofectamine 2.0 was used with the siRNA. The administration was intravenously retroocularly, 24 hours before, or 24 hours and 72 hours after infection. A 15-day survival was observed in the group that received the siRNA compared to the control group with an average of 5 days [46]. For West Nile virus (WNV), two siRNAs were designed, one directed to the NS2B region and another to the NS5, in addition to a construction of a bifunctional siRNA joining the motif 5′-UGUGU-3′ at the end of both siRNAs (NSA2A-is siRNA and NS5-is siRNA). An *in vitro* test was performed on Vero cells at different concentrations of siRNA (5-100 nM), transfecting them with the siPORT Amine reagent for 4 h, and subsequently, viral infection was performed at an MOI of 0.1 for 90 minutes, resulting in a partial inhibition of the virus at 20 nM of the siRNA directed at NS5, 100 nM for the siRNA directed at NS2A, and 10 nM for NSA2A-is siRNA and NS5-is siRNA, with a reduction with these siRNAs of 98.99% of the viral titer at 48 hpi [47]. Previous trials showed positive effects towards the protection of cells transfected with siRNAs and subsequently infected with the virus, as well as preventing postinfection viral replication. It will be interesting to analyze the effect of our siRNA using an *in vivo* model such as a mosquito vector. This approach will be a successful tool to avoid ZIKV transmission.

siRNA specificity was conducted with other arboviruses, one flavivirus (DENV) and an alphavirus (CHIKV). As it is shown in Table 1, CT values did not show a significant change with these viruses indicating that the siRNA was specific to ZIKV, probably because the siRNA is directed to a sequence present in the 3′UTR that is not shared by other close-related viruses. However, since 3′UTR is a conserved sequence among flaviviruses, it will be interesting to design a siRNA directed against a common sequence present in several important flaviviruses such as yellow fever virus (YFV), WNV, DENV, and Japanese Encephalitis virus (JEV) 323.

## 4. Conclusion

Transfection of 1 and 2 ug of siRNA was able to reduce CTs significantly (*p* < 0.05) in C6/36 cells after infection with ZIKV at a MOI of 0.001 for 1 hour. Its effect is specific for ZIKV and not for other arboviruses such as DENV and CHIKV. This siRNA could be considered a potential therapeutic towards the disease that causes ZIKV and the medical problems generated.

## Figures and Tables

**Table 1:** Quantity RT-qPCR values of ZIKV, DENV, and CHIKV after siRNA transfection.

	Negative control	Positive control	siRNA (0.5 *μ*g)	siRNA (1 *μ*g)	siRNA (2 *μ*g)
*ZIKV*					
CT	—	22.33 ± 0.43	24.56 ± 0.77	28.24 ± 0.34^∗^	33.73 ± 1.82^∗^
*Δ*CT	—	—	2.23	5.91	10.11
*DENV*					
CT	—	25.99 ± 1.18	24.78 ± 0.84	26.90 ± 1.13	27.06 ± 0.38
*Δ*CT	—	—	1.21	0.93	1.06
*CHIKV*					
CT	—	14 ± 0.81	14	14	13.66 ± 0.94
*Δ*CT	—	—	0	0	0.34

^∗^Statistical significance (*p* < 0.05).

## Data Availability

All data generated or analyzed during this study are included in the published article. The raw data files are available upon request to the corresponding author.
